# Mutational analysis of *Arabidopsis thaliana* ABCE2 identifies important motifs for its RNA silencing suppressor function

**DOI:** 10.1111/plb.13193

**Published:** 2020-11-29

**Authors:** J. Mõttus, S. Maiste, P. Eek, E. Truve, C. Sarmiento

**Affiliations:** ^1^ Department of Chemistry and Biotechnology Tallinn University of Technology Tallinn Estonia

**Keywords:** ABCE1, ABCE2, AtRLI2, RNA silencing, suppression of RNA silencing, *Arabidopsis thaliana*

## Abstract

- ATP‐binding cassette sub‐family E member 1 (ABCE1) is recognized as a strongly conserved ribosome recycling factor, indispensable for translation in archaea and eukaryotes, however, its role in plants remains largely unidentified. *Arabidopsis thaliana* encodes two paralogous ABCE proteins (AtABCE1 and AtABCE2), sharing 81% identity. We previously reported that AtABCE2 functions as a suppressor of RNA silencing and that its gene is ubiquitously expressed. Here we describe the structural requirements of AtABCE2 for its suppressor function.
- Using agroinfiltration assays, we transiently overexpressed mutated versions of AtABCE2 together with GFP, to induce silencing in GFP transgenic *Nicotiana benthamiana* leaves. The influence of mutations was analysed at both local and systemic levels by *in vivo* imaging of GFP, Northern blot analysis of GFP siRNAs and observation of plants under UV light.
- Mutants of AtABCE2 with impaired ATP binding in either active site I or II failed to suppress GFP RNA silencing. Mutations disrupting ATP hydrolysis influenced the suppression of silencing differently at active site I or II. We also found that the N‐terminal iron–sulphur cluster domain of AtABCE2 is crucial for its suppressor function.
- Meaningfully, the observed structural requirements of AtABCE2 for RNA silencing suppression were found to be similar to those of archaeal ABCE1 needed for ribosome recycling. AtABCE2 might therefore suppress RNA silencing *via* supporting the competing RNA degradation mechanisms associated with ribosome recycling.

## Graphical abstract

ATP binding and hydrolysis, mediated by Walker A and B motifs, are important for *Arabidopsis thaliana* ABCE2 RNA silencing suppressor function and so is its FeS cluster domain.

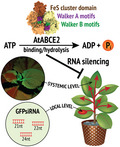

## INTRODUCTION

ATP‐binding cassette sub‐family E member 1 (ABCE1) is a multi‐functional protein, which is highly conserved among eukaryotes and archaea. Inactivation of the *ABCE1* gene in all organisms tested so far leads to lethality or to severe morphological alterations (Petersen *et al.,* 2004; Kispal *et al.,* 2005; Navarro‐Quiles *et al.,* 2018). Initially, ABCE1 was described in mammals as RNase L Inhibitor (RLI), a component of the 2‐5A system, an antiviral pathway induced by interferons (Bisbal *et al.,* 1995). Blocking RNase L, ABCE1 inhibits viral mRNA degradation and regulates cellular mRNA stability in mammalian cells (Le Roy *et al.,* 2001; Salehzada *et al.,* 2009). Extensive studies in recent years have demonstrated that ABCE1 is associated with different stages of protein synthesis in eukaryotes (Navarro‐Quiles *et al.,* 2018). In yeast, ABCE1 interacts with eukaryotic initiation factors eIF5, eIF2 and eIF3 and promotes the assembly of the pre‐initiation complex (Dong *et al.,* 2004; Chen *et al.,* 2006; Toompuu *et al.,* 2016). Moreover, ABCE1 is involved in ribosome biogenesis and transport in yeast. Indeed, it was demonstrated that in yeast and mammalian cells ABCE1 is localized in both cytoplasm and nucleus (Kispal *et al.,* 2005; Yarunin *et al.,* 2005; Toompuu *et al.,* 2016). However, the central role of ABCE1 in eukaryotes and archaea is in ribosome recycling, the process linking translation termination with initiation. Together with release factor 1 (eRF1/aRF1), ABCE1 promotes the dissociation of the post‐termination complex, enabling translation re‐initiation (Pisarev *et al.,* 2010; Barthelme *et al.,* 2011; Nürenberg & Tampé, 2013; Nürenberg‐Goloub *et al.,* 2018). Besides its role in canonical translation termination, ABCE1 together with eRF1/eRF3 paralogues Pelota and Hbs1 splits stalled ribosomes, activating mRNA surveillance pathways, namely, nonstop and no‐go decay. These RNA decay mechanisms degrade aberrant mRNAs, lacking stop codons or containing elongation inhibiting features (Pisareva *et al.,* 2011; Kashima *et al.,* 2014; Hashimoto *et al.,* 2017; Navarro‐Quiles *et al.,* 2018). Thus, data suggest a pivotal role of ABCE1 in RNA homeostasis.

Although, most genomes encode a single ABCE protein, in some species two or more *ABCE* genes were identified (Navarro‐Quiles *et al.,* 2018). *Arabidopsis thaliana* encodes two highly similar *ABCE* paralogues: *AtABCE1* and *AtABCE2*. According to the transcriptome map, based on RNA‐seq (Klepikova *et al.,* 2016), *AtABCE1* expression is restricted to generative organs, while *AtABCE2* is expressed in all tissues, suggesting its global role throughout the plant’s life. We have previously reported that AtABCE2, also named AtRLI2, as well as its human orthologue ABCE1 function as suppressors of RNA silencing, another RNA degradation mechanism, which is conserved among eukaryotes (Sarmiento *et al.,* 2006; Kärblane *et al.,* 2015). Although there is growing data concerning the role of ABCE1 in translation and mRNA surveillance, there is no information about the precise activity of ABCE proteins in RNA silencing suppression.

RNA silencing is triggered by double‐stranded RNA (dsRNA). In plants, dsRNA is processed by Dicer‐like enzymes into small RNA (sRNA) duplexes, ranging from 21 to 24 bp in size. Loaded into an Argonaute (AGO) containing RNA‐induced silencing complex (RISC), sRNA guide RISC for degradation of complementary RNA or inhibition of its translation (Hammond *et al.,* 2000; Hamilton *et al.,* 2002; Baulcombe, 2004; Brodersen & Voinnet, 2006). RNA silencing can be amplified in plants through the action of RNA‐dependent RNA polymerase (RDR), giving rise to secondary small interfering RNAs (siRNA). Moreover, RNA silencing may spread from cell to cell *via* plasmodesmata, or systemically through the phloem beyond the initiation site and guide the target degradation in distant tissues (Tang *et al.,* 2003; Brosnan *et al.,* 2007; Pyott & Molnár, 2015; Zhang *et al.,* 2019). Through different pathways, RNA silencing mediates developmental gene regulation, and biotic and abiotic stress responses (Ding & Voinnet, 2007; Sunkar *et al.,* 2007). Furthermore, this mechanism plays a major role in antiviral immunity in plants. In this context, amplification and spread of RNA silencing are beneficial for the host and, thus, it is not surprising that viruses encode RNA silencing suppressors (Baulcombe, 2004; Burgyán & Havelda, 2011). In addition, plants also encode suppressors to regulate the expression of endogenous genes. Only a few of them, including AtABCE2, have been identified so far (Gazzani *et al.,* 2004; Sarmiento *et al.,* 2006; Gy *et al.,* 2007; Yu *et al.,* 2015; Zhang *et al.,* 2015; Li *et al.,* 2017; Huang *et al.,* 2019). Interestingly, essential components of mRNA surveillance pathways were found among plant endogenous RNA silencing suppressors, supporting an obvious connection between these RNA degradation mechanisms (Gazzani *et al.,* 2004; Gy *et al.,* 2007; Yu *et al.,* 2015; Zhang *et al.,* 2015). We previously demonstrated that AtABCE2 affects the accumulation of siRNAs when overexpressed (Sarmiento *et al.,* 2006). In this research, we aimed to assess the structural requirements for AtABCE2 suppressor function.

The ABCE proteins belong to the large ATP‐binding cassette (ABC) protein superfamily. They contain two nucleotide binding domains (NBD1 and NBD2) characteristic for ABC proteins, which compose the core for ATP binding and hydrolysis (Braz *et al.,* 2004). In addition, ABCE proteins possess several specific domains important for their diverse functions, including the ‘hinge’ domain and N‐terminal iron‐sulphur (FeS) cluster domain. The crystal structure of archaeal ABCE1 (aABCE1) shows that both NBDs, arranged by ‘hinge’ in a head‐to‐tail orientation, compose the V‐like cavity with two ATP‐binding sites in the interface. Each active site is composed of highly conserved Walker A (WA) and Walker B (WB) motifs from one NBD and C‐signature from the opposite one. The WA motif from one NBD binds α‐ and β‐phosphates of the ATP molecule, while C‐signature from the other NBD binds γ‐phosphate of the same ATP molecule. Consequently, ATP hydrolysis relies on the WB located to the same NBD as the WA motif (Braz *et al.,* 2004; Karcher *et al.,* 2005). Because of high amino acid sequence conservation among all ABCE proteins, the structures of eukaryotic ABCEs can be deduced from those of aABCE1 (Navarro‐Quiles *et al.,* 2018). It was suggested that ATP binding and hydrolysis in aABCE1 induces tweezer‐like motion of the NBD‐dimer, which in turn induces conformational changes in the associated domains that are transmitted to the interaction partners (Karcher *et al.,* 2005; Karcher *et al.,* 2008). Recent research on aABCE1 as ribosome recycling factor confirmed the proposed model and described the action of aABCE1 in detail. According to the results, ABCE1 binds to the post‐termination complex in a semi‐closed state with one ATP molecule occluded in the second ATP‐binding site (site II). The occlusion of both ATP molecules causes radical relocation of the FeS cluster domain towards the cleft between ribosomal subunits, and this leads to ribosome splitting. After formation of the post‐splitting complex and recruitment of initiation factors, aABCE1 dissociates from the small ribosomal subunit upon hydrolysis of ATP. Interestingly, hydrolysis of ATP at site II, but not at site I, is crucial at this step (Nürenberg‐Goloub *et al.,* 2018). In addition, it was previously demonstrated that mutants of the catalytic site I display reduced ATPase activity, while inactivation of site II makes aABCE1 hyperactive. These studies revealed the functional asymmetry of the two ATP binding sites in ATP hydrolysis, ribosome binding and splitting (Barthelme *et al.,* 2011; Nürenberg‐Goloub *et al.,* 2018). In addition, helix‐loop‐helix (HLH) insertion in the NBD1, with earlier unclear function, was shown to be an important site for interaction with the ribosomal small subunit in the post‐splitting complex (40S/30S·ABCE1) (Kiosze‐Becker *et al.,* 2016; Heuer *et al.,* 2017; Nürenberg‐Goloub *et al.,* 2020). ABCE1 can remain bound to the small ribosomal subunit until the final step of translation initiation, preventing it from re‐association with the large ribosomal subunit (Simonetti *et al.,* 2020).

In order to understand how AtABCE2 suppresses RNA silencing, we carried out mutational analysis of this protein and tested it in GFP‐transgenic *Nicotiana benthamiana* plants. Mutants impaired in ATP binding or ATP hydrolysis or lacking potential interaction sites (FeS cluster domain or HLH) were assessed for their ability to suppress GFP transgene RNA silencing at the local and systemic levels.

## MATERIAL AND METHODS

### Cloning of AtABCE2 mutants

To obtain AtABCE2 mutants the construct pBin61‐AtABCE2 (named in a previous publication as pBin61‐AtRLI2) was used as the primary template (Kärblane *et al.,* 2015). Mutants bearing single amino acid substitutions (WB1, WB2, WA1 and WA2) and that carrying the HLH motif deletion (AtABCE2∆HLH) were generated by two‐step PCR amplification. In the first step, two fragments of AtABCE2 were amplified by Phusion polymerase and subsequently ligated by T4 DNA ligase (Thermo Fisher Scientific, Waltham, MA, USA), according to the manufacturer’s protocol. The first fragment was produced using 5′‐AtABCE2 and 3′‐PR1 primers. The second fragment was obtained with primers 5′‐PR2 and 3′‐AtABCE2. Finally, using 5′‐AtABCE2 and 3′‐AtABCE2 primers, the full‐length AtABCE2 mutated cDNAs were obtained. Since AtABCE2∆FeS implies the deletion of the 5′ terminus of AtABCE2 cDNA, mutated cDNA was obtained by one‐step PCR amplification. All mentioned mutants were cloned into Invitrogen^TM^ pcDNA3.1/V5‐His TOPO vector according to the manufacturer’s manual (Thermo Fisher Scientific) and transformed into TOP10 *E. coli* cells. In parallel, the AtABCE2 sequence was also cloned into pcDNA3.1/V5‐His TOPO vector. WA1C2, WA2C1 and WB1/2 were generated with QuikChange II XL Site‐Directed Mutagenesis Kit (SDM) (Agilent Technologies, Santa Clara, CA, USA), using as templates pcDNA3.1‐WA1‐V5, pcDNA3.1‐WA2‐V5 and pcDNA3.1‐WB1‐V5 constructs, respectively, following the manufacturer’s instructions. To produce the WA1/2C1/2 mutant, point mutations in WA2 and C‐signature 1 were introduced into pcDNA3.1‐WA1C2‐V5 by two subsequent SDM procedures. Primers used in mutagenesis are listed in Table 1.

**Table 1:** List of primers used to generate AtABCE2 wild type and AtABCE2 mutated sequences. Substituted nucleotides are underlined in the case of mutagenizing primers.

primer name	primer sequence (5′–3′)	cloning of construct(s)	mutation(s) obtained
3′‐PR1WA1	CTC TCC AAT ACC ATT GGT TCC A	WA1	K116E
5′‐PR2WA1	TCA ACT GCT CTG AAA ATT		
3′‐PR1WA2	TCT CCC CTG TAC CAT TCT	WA2	K387E
5′‐PR2WA2	CAA CAT TTA TTC GGA TGC T		
3′‐PR1WB1	ATC GAG GTA ACT AGA TGG TTG AT	WB1	E242Q
5′‐PR2WB1	GTT AAG CAA AGA CTC AAA GCT		
3′‐PR1WB2	CGA GAT ATG CAC TTG GCT GAT	WB2	E493Q
5′‐PR2WB2	ATT CTG AGC AAC GTA TTG TT		
3′‐PR1∆HLH	GGA GGA CTA GTG AAA CGG CCC AAA T	AtABCE2∆HLH	∆D139‐L160
5′‐PR2∆HLH	AGA AGA TAA TCT TAA GGC CAT T		
5′‐PR2∆FeS 3′‐AtABCE2	CAC CAT GAT CAA TCT TCC A ATC ATC CAA GTA GTA GTA TGA G	AtABCE2∆FeS	∆A2‐I72
5′‐AtABCE2 3′‐AtABCE2	CAC CAT GGC AGA TCG ATT G ATC ATC CAA GTA GTA GTA TGA G	AtABCE2 wt and the above listed mutants	
SDM fwC2	TCA AGA AGT TGT CAA TCT CCG AGG AGG AGA ATT GCA AAG GG	WA1C2	K116G, S469R
SDM revC2	CCC TTT GCA ATT CTC CTC CTC GGA GAT TGA CAA CTT CTT GA		
SDM fwC1	CGT GAT GTT GAG AAT TTA CGT GGT GGT GAG CTG CAG AG	WA2C1	K387G, S218R
SDM revC1	CTC TGC AGC TCA CCA CCA CGT AAA TTC TCA ACA TCA CG		
SDM fw1C1	CGT GAT GTT GAG AAT TTA CGT GGT GGT GAG CTG CAG AG	WA12C12	K116G, S469R, K387G, S218R
SDM rev1C1	CTC TGC AGC TCA CCA CCA CGT AAA TTC TCA ACA TCA CG		
SDM fw2C2	TCA AGA AGT TGT CAA TCT CCG AGG AGG AGA ATT GCA AAG GG		
SDM rev2C2	CCC TTT GCA ATT CTC CTC CTC GGA GAT TGA CAA CTT CTT GA		
SDM fwWB2	GCC TGC GGA TAT ATA CCT GAT CGA TCA GCC AAG TGC ATA TCT CG	WB12	E242Q, E493Q
SDM revWB2	CGA GAT ATG CAC TTG GCT GAT CGA TCA GGT ATA TAT CCG CAG GC		

For agroinfiltration assays, sequences of AtABCE2 wild type (wt) and its mutants, all containing the V5‐His‐tag, were excised from pcDNA3.1/V5‐His TOPO vectors with *Kpn*I and *Mss*I restriction enzymes and cloned into the binary plasmid pBin61 downstream of Cauliflower mosaic virus (CaMV) 35S promoter as described (Kärblane *et al.,* 2015). The obtained constructs were sequenced and transformed into *Agrobacterium tumefaciens* strain C58C1 harbouring pCH32 (Hamilton *et al.,* 1996).

### Plant material and growth conditions

Seeds of *N. benthamiana* stably expressing the GFP gene (16c line) were kindly provided by David Baulcombe. Plants were grown in a plant chamber at 25 °C with a 16‐h photoperiod. Plants used for agroinfiltration were 4–5 weeks old.

### Agroinfiltration assay and GFP imaging

All recombinant *A. tumefaciens* cultures were prepared for infiltration as described in Hamilton *et al.* (2002) with several modifications. Overnight cultures were used to inoculate 20 ml of the final medium: LB broth medium supplemented with kanamycin (50 μg ml^−1^), tetracycline (5 μg ml^−1^), 10 mm 2‐(N‐morpholino)ethanesulfonic acid (MES, pH 6.3) and 20 μm acetosyringone. After overnight incubation (29 °C, 180 rpm), cells were collected by centrifugation (3,000 *g* for 5 min at room temperature), washed once with distilled water (3,000 *g* for 5 min at room temperature) and resuspended in the infiltration buffer (10 mm MES, 150 μm acetosyringone and 10 mm MgCl_2_). All suspensions were adjusted to a final OD_600_ of 0.5 and incubated with slow shaking for 3 h. *A. tumefaciens* carrying the inducer of GFP RNA silencing (GFP gene cloned into pBin61) was mixed in 1:1 ratio with *A. tumefaciens* harbouring the empty vector pBin61 or the vector containing either AtABCE2 wt or one of its mutated sequences. The agrobacteria mixtures were named GFP/pBin61, GFP/AtABCE2 and GFP/AtABCE2‐mutant, respectively. The pBin61 vector and agrobacteria containing GFP in pBin61 were kindly provided by D. Baulcombe.

For local silencing suppression assays, leaves of 16c *N. benthamiana* were infiltrated with GFP/pBin61 and GFP/AtABCE2 or with GFP/AtABCE2‐mutant mixtures into opposite sides of the leaf midrib. Because of high variability in GFP fluorescence intensity between leaves, the levels of GFP expression were always compared separately between infiltrated patches in each leaf. At 5 dpi, GFP fluorescence was quantified and analysed using IVIS Lumina II (Caliper Life Sciences, Waltham, MA, USA) and Living Image (version 4.1) software as described in Stephan *et al.* (2011) including the modifications reported in Kärblane *et al.* (2015). For the local silencing suppression assay, we measured radiant efficiency of GFP in agroinfiltrated leaves and analysed the difference between two patches in the same leaf using a paired two‐tail *t*‐test. To assess suppression of systemic silencing, five to seven plants were infiltrated with each agrobacteria mixture and the spread of GFP silencing to the upper leaves was followed over 3 weeks. GFP fluorescence was observed under long wavelength ultraviolet (UV) light (Black‐Ray B‐100AP, Ultraviolet Products , Analytik Jena US, Upland, CA, USA) at 14 and 21 dpi. GFP systemic silencing can be seen as the appearance of red tissues in new growing leaves of the infiltrated plants. If more than 90% of non‐infiltrated leaves of a plant remained green, the suppression of systemic silencing was evidenced (Jing *et al.,* 2011). At 14 dpi, plants were photographed with a Nikon p7000 camera (Nikon, Tokyo, Japan) using a yellow filter (Tokina, Tokyo, Japan). The data from nine independent experiments (for each AtABCE2 mutant, four to six experiments were carried out) were pooled and analysed with a Chi‐square test and Fisher's exact test as post‐test (Tables S1, S2). AtABCE2 and its mutants were compared to pBin61 in a pairwise manner. In all tests *P* ≤ 0.05 was considered statistically significant. Statistical analysis was carried out using JMP 14 software (SAS Institute, Cary, NC, USA).

### Extraction of RNA and Northern blot analysis

Total RNA was isolated from infiltrated leaf patches, collected at 5 dpi, using Invitrogen TRIzol^TM^ reagent (Thermo Fisher Scientific) following the manufacturer’s instructions. A total of 25 μg of total RNA were resolved in 15% denaturing polyacrylamide gel (8 m urea) as previously described (Olspert *et al.,* 2014). After electrophoresis, the gel was cut in two: the lower part was used for analysis of GFP siRNA and the upper part for U6 snRNA, utilized as loading control. RNA was transferred to Amersham Hybond^TM^‐N + membrane (GE Healthcare, Chicago, IL, USA) by overnight electroblotting in 0.5 × TBE at constant current (450 mA) and fixed to the membrane by UV cross‐linking twice at 1,200 mJ (Stratagene UV Stratalinker 1800). To detect GFP‐specific siRNAs, the [α‐^32^P] UTP‐labelled *in vitro* transcript corresponding to anti‐sense GFP was synthesized (MAXIscript kit, Thermo Fisher Scientific) according to the manufacturer’s protocol. For loading control, DNA oligos complementary to U6 snRNA were end‐labelled with [γ‐^32^P] ATP using T4 polynucleotide kinase (Thermo Fisher Scientific). Radioactive probes were purified through NICK Sephadex G‐50 columns (GE Healthcare). Hybridization and washing conditions were carried out as previously described (Szittya *et al.,* 2002), with the following modifications for U6 detection: ULTRAhyb‐Oligo buffer (Thermo Fisher Scientific) was used and an additional wash under more stringent conditions was performed. Radioactive signals were scanned by Personal Molecular Imager FX (BioRad, Hercules, CA, USA) after 30 min exposure for U6 and 24 h for siRNAs. Bands of interest (GFP siRNAs) were quantified by densitometry using ImageQuant TL software (GE Healthcare), normalized to U6 bands and always comparing each construct *versus* pBin61.

### Protein structure modelling

The amino acid sequence of AtABCE2 was provided as input for the HHpred server (Zimmermann *et al.,* 2018) to search for models of homologous proteins. Default parameters were used. Three models (PDB IDs: 3BK7, 1YQT and 3OZX) were selected as templates and the corresponding alignment was submitted to MODELLER (Webb & Sali, 2016) for comparative modelling. Molecular graphics were produced in UCSF ChimeraX (Goddard *et al.,* 2018).

## RESULTS

### Suppression of GFP RNA silencing by WA1C2, WA2C1 and WA12C12 mutants

**Fig. 1:**
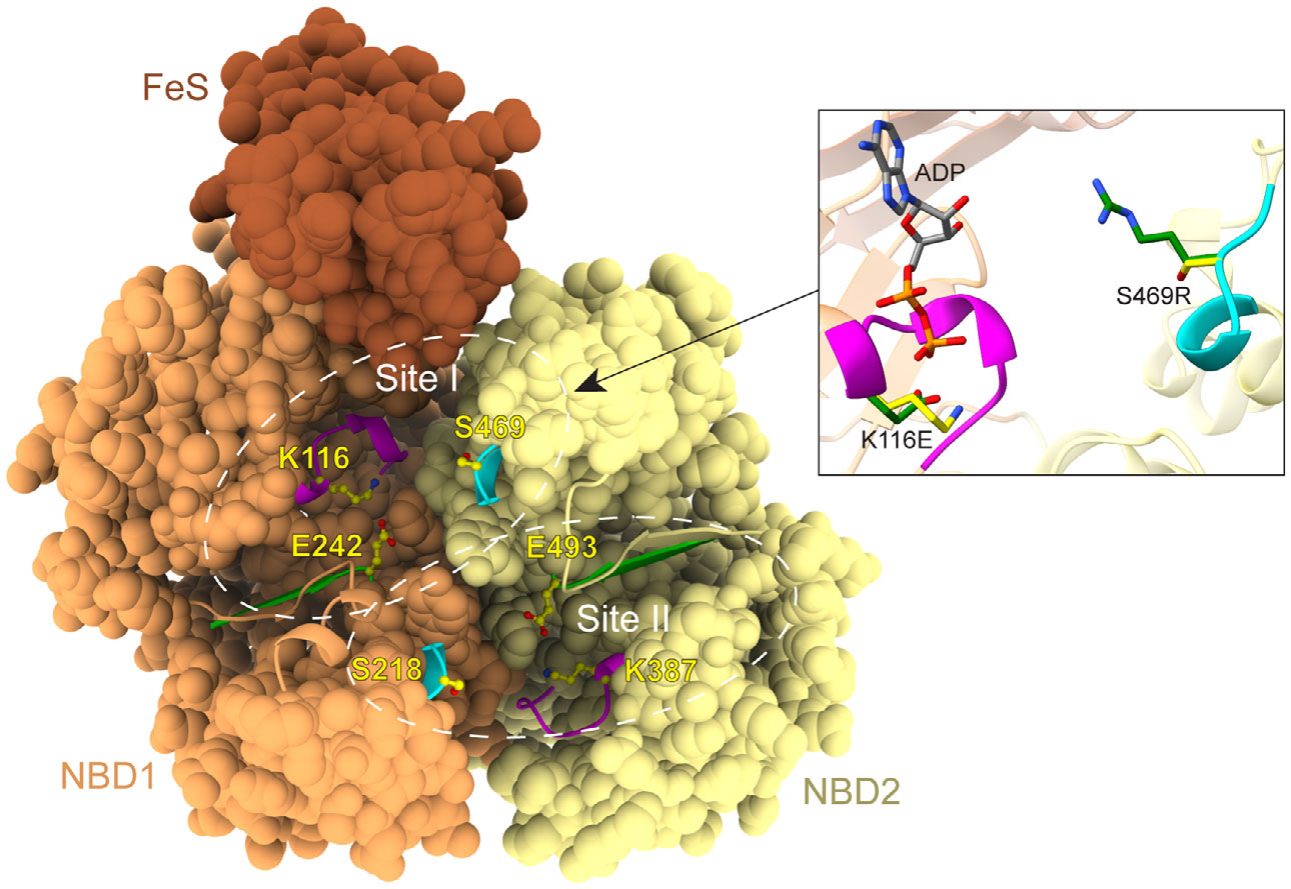
Homology model of AtABCE2. The FeS cluster domain (FeS) and the two nucleotide‐binding domains (NBD1 and NBD2) are rendered as space‐filling models. Conserved motifs Walker A (WA, magenta), Walker B (WB, green) and C‐signature (cyan) are highlighted as ribbons in both binding sites, with the residues of interest modelled as yellow balls and sticks. Inset: The mutations introduced to ATP binding site I in this study are illustrated using stick representation (wild type residues in yellow, mutant in green) with an ADP molecule aligned as it is bound in the crystal structure model of *Pyrococcus abysii* ABCE1 (PDB ID 3BK7). Oxygen atoms are in red, nitrogen in blue and phosphorus in orange.

Based on mutational analysis carried out earlier in ABCE1 from archaea and yeast (Karcher *et al.,* 2008; Barthelme *et al.,* 2011), we generated AtABCE2 analogous mutants (Table 1). First, we questioned whether ATP binding by AtABCE2 is needed for the suppression of RNA silencing. Taking into account high conservation of ABCE proteins and numerous evidence on the structural requirements for ATP binding by ABC superfamily, as well as by aABCE1 proteins, we assume that the key residues play the same role for AtABCE2 (ter Beek *et al.,* 2014; Navarro‐Quiles *et al.,* 2018; Nürenberg‐Goloub *et al.,* 2018). The mutant with assumed affected ATP binding in the first active site (site I) contained substitutions K116E in the WA motif from NBD1 and S469R in the C‐signature from NBD2 (from here on named WA1C2 mutant). The corresponding residues were mutated in WA2 and C1 motifs (substitutions K387E and S218R, respectively) to obtain the mutant WA2C1 with affected ATP binding in the second active site (site II). In addition, we generated the AtABCE2 mutant lacking ATP binding activity at both sites (named WA12C12). ATP binding by AtABCE2 is illustrated by the homology model based on published aABCE1 crystal structures (Fig. 1).

**Fig. 2:**
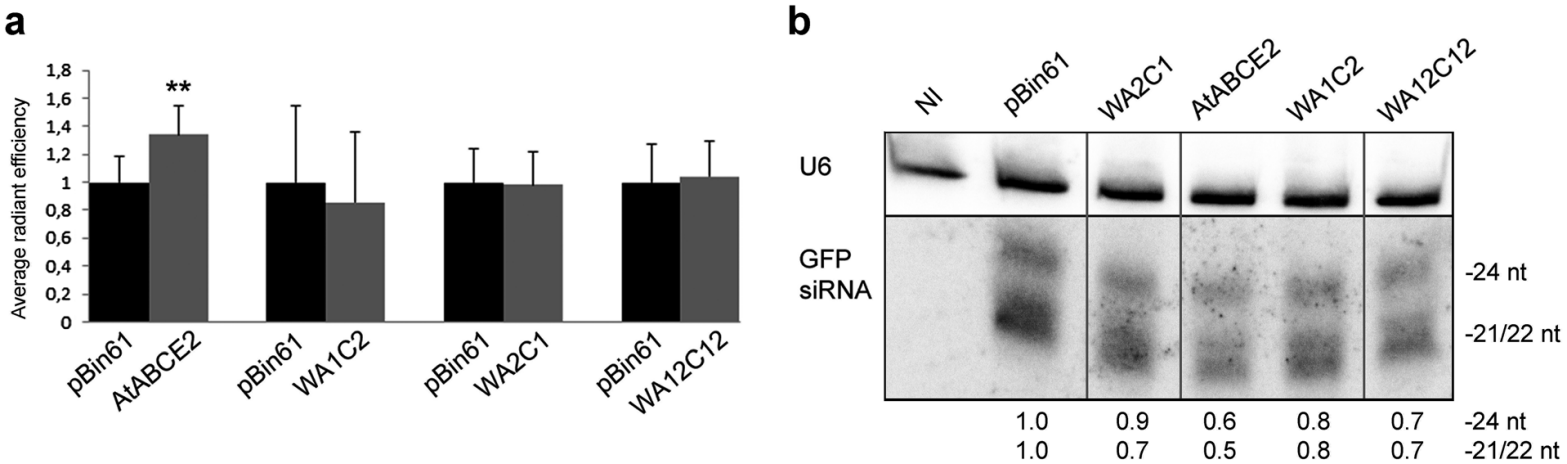
Suppression of local GFP RNA silencing by WA1C2, WA2C1 and WA12C12 mutants. *Agrobacterium tumefaciens* harbouring pBin61‐GFP was infiltrated together with *A. tumefaciens* carrying pBin61‐AtABCE2 mutant or pBin61‐AtABCE2 wt into one side of GFP transgenic *Nicotiana benthamiana* (16c) leaf midrib (indicated as WA1C2, WA2C1, WA12C12 and AtABCE2). The other half of the leaf was infiltrated with GFP/pBin61 agrobacteria mixture (indicated as pBin61). At 5 dpi, GFP fluorescence in the infiltrated patches was detected and quantified using an *in vivo* imaging system. (a) The means of GFP radiant efficiency were compared between pBin61 and AtABCE2 (n = 25), pBin61 and WA1C2 (n = 27), pBin61 and WA2C1 (n = 28), pBin61 and WA12C12 (n = 27). Statistical analysis was performed using a paired two‐tail *t‐*test (**P < 0.01). Standard errors are indicated as error bars. (b) Total RNA was extracted from agroinfiltrated patches and analysed by Northern blot. GFP‐specific siRNAs were detected using [α‐^32^P] UTP‐labelled antisense GFP transcript. As loading control, U6 snRNA was detected with [γ‐^32^P] ATP‐labelled complementary DNA oligos. The levels of GFP siRNAs (24 and 21/22 nt long), quantified with ImageQuant TL, were normalized to U6 and compared with pBin61. NI indicates non‐infiltrated leaf. For better visualisation, the lanes were cropped from the same blots. Intact membranes are presented in Figure S2a.

To evaluate the suppression activity, we transiently overexpressed the generated mutants together with an RNA silencing inducer (GFP gene) in the leaves of GFP‐transgenic *N. benthamiana* plants (16c line). For local silencing suppression assays, we infiltrated *A. tumefaciens* carrying the GFP gene cloned into pBin61 together with *A. tumefaciens* harbouring empty vector pBin61 (GFP/pBin61) into one side of the leaf midrib and *A. tumefaciens* harbouring GFP together with *A. tumefaciens* carrying either AtABCE2 wt or the mutated sequence (GFP/AtABCE2 wt or GFP/AtABCE2 mutant) into the other side of the midrib. Normally, after 2 days post‐infiltration (dpi), the GFP expression in the infiltrated patches started to decline due to the initiation of RNA silencing by the endogenous plant machinery. However, in the case of suppression, we observed elevated GFP expression levels also after 2 dpi. Using an *in vivo* imaging system, equipped with a GFP‐specific filter set, we detected GFP fluorescence and measured its radiant efficiency in both infiltrated patches of each leaf at 5 dpi. GFP radiant efficiency values obtained from infiltrated patches were normalized to the background and compared in each leaf separately (Figure S1). As expected, in the presence of AtABCE2, GFP fluorescence levels were higher compared to the empty plasmid pBin61 (*P* = 0.002), confirming the suppressor activity. In contrast, there was no significant difference between any of the mutants (WA1C2, WA2C1 and WA12C12) and pBin61, indicating that these mutants are unable to suppress local silencing (Fig. 2a).

To confirm the results at the molecular level, we collected the infiltrated tissues and performed Northern blot analysis of GFP‐specific siRNAs. In the patches infiltrated with GFP/pBin61 agrobacteria mixture, GFP siRNAs of all size classes were abundant. AtABCE2, in turn, strongly reduced accumulation of siRNAs. In the case of WA1C2, WA2C1 and WA12C12 mutants, siRNA levels were higher compared to AtABCE2 wt. This is in agreement with the observed decline of GFP fluorescence levels in the infiltrated patches (Fig. 2b).

**Fig. 3:**
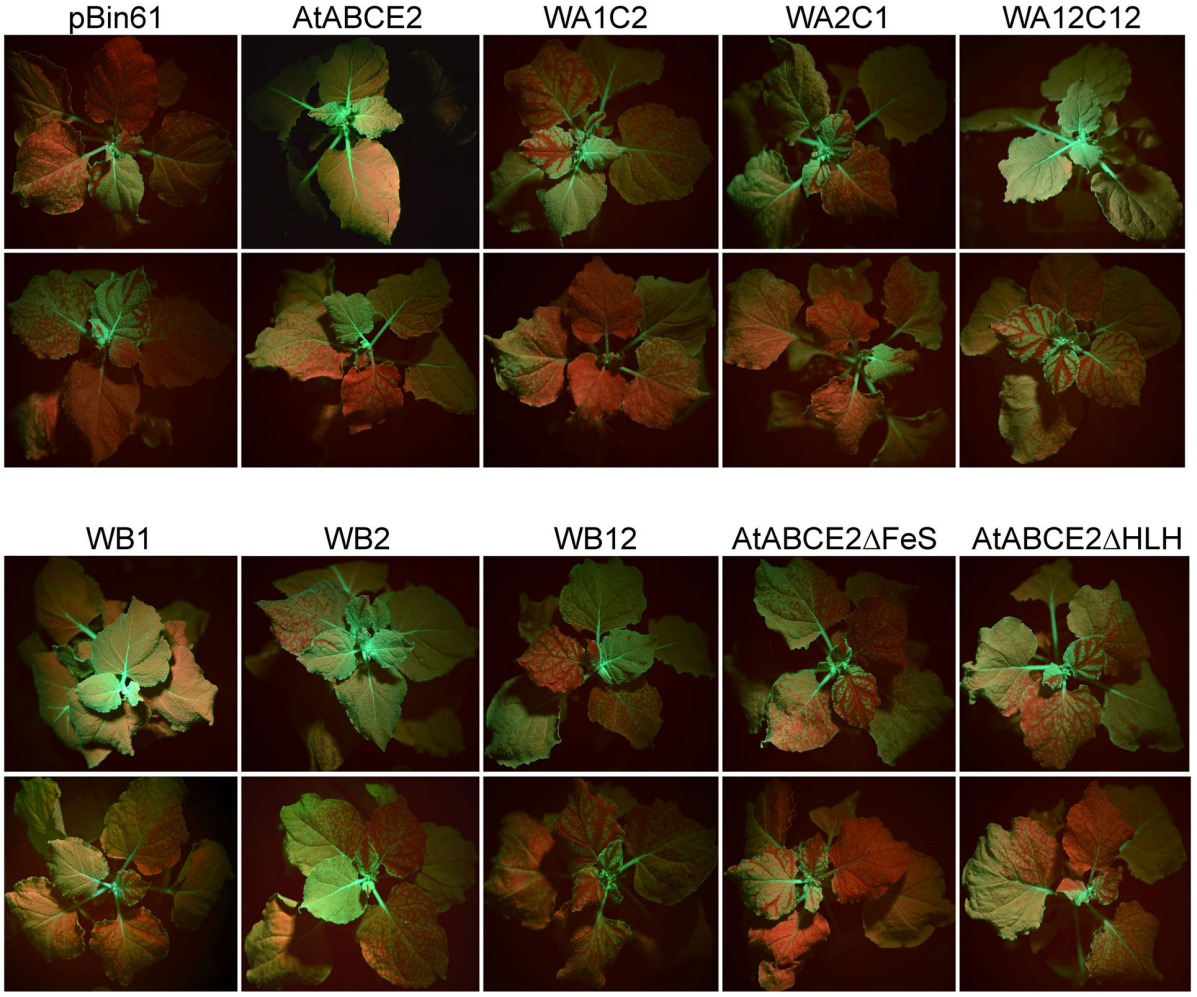
Suppression of systemic GFP RNA silencing by AtABCE2 mutants. Four‐ to 5‐week‐old GFP‐transgenic *Nicotiana benthamiana* plants were co‐infiltrated with *Agrobacterium tumefaciens* harbouring pBin61‐GFP and pBin61‐AtABCE2 wt or pBin61‐AtABCE2 mutant or pBin61 (indicated as AtABCE2, WA1C2, WA2C1, WA12C12, WB1, WB2, WB12, AtABCE2∆FeS, AtABCE2∆HLH and pBin61). Representative plants were photographed under UV light at 14 dpi. The red tissues in the leaves indicate systemic RNA silencing of GFP.

We further analysed the suppression of systemic GFP RNA silencing by the mutants. For this purpose, we infiltrated *N. benthamiana* leaves as indicated above and monitored the spread of GFP silencing in whole plants over the following 3 weeks. Systemic GFP silencing can be visualized under UV light as emerging red tissue in the new, growing leaves. The percentages of systemically silenced plants are shown in Table 2. In the case of AtABCE2 mutants, the percentages were calculated taking into account four to six independent experiments, each including five to seven plants (Table S1). Almost all plants infiltrated with GFP/pBin61 showed extensive spread of GFP systemic silencing (91% at 14 dpi and 95% at 21 dpi), while in the presence of AtABCE2, more than half of the plants had over 90% completely green, non‐infiltrated leaves, indicating suppression of systemic silencing, as reported previously (Sarmiento *et al.,* 2006). At 14 dpi, 44% of the plants infiltrated with AtABCE2 wt were silenced and 1 week later the percentage rose to 47%. Over 70% of the plants infiltrated with the mutants WA1C2 or WA2C1 showed systemic silencing at 14 and 21 dpi, meaning that this mutations affected the suppressor function of AtABCE2. In contrast, the amount of plants infiltrated with GFP/WA12C12 displaying systemic silencing was less than that of the previous mutants (53–55%) and showed a statistically significant difference from the empty vector (Fig. 3, Table 2). Therefore, mutations in both WA and both C‐signature motifs seem to retain the suppressor function of AtABCE2 at the systemic level.

**Table 2:** Percentage of plants showing systemic silencing at two different time points. The number of plants displaying systemic silencing out of the total number of infiltrated plants is provided in brackets.

construct	14 dpi	21 dpi
GFP + pBin61	91% (50/55)	95% (52/55)
GFP + AtABCE2	44%*** (24/55)	47%*** (26/55)
GFP + WA1C2	71% (17/24)	79% (19/24)
GFP + WA2C1	75% (18/24)	83% (20/24)
GFP + WA12C12	53%** (20/38)	55%*** (21/38)
GFP + WB1	56%** (19/34)	56%*** (19/34)
GFP + WB2	71%* (24/34)	79% (27/34)
GFP + WB12	79% (19/24)	79% (19/24)
GFP + AtABCE2∆FeS	76% (26/34)	79% (27/34)
GFP + AtABCE2∆HLH	79% (23/29)	83% (24/29)

*** Statistically significant difference with GFP + pBin61, used as a control group (*P* < 0.0001).

** Statistically significant difference with GFP + pBin61, used as a control group (*P* < 0.001).

* Statistically significant difference with GFP + pBin61, used as a control group (*P* < 0.05).

Our results show that ATP binding at active centre I and II is necessary for the suppression activity of AtABCE2, at least at the local level.

### Suppression of GFP RNA silencing by WB1, WB2 and WB12 mutants

**Fig. 4:**
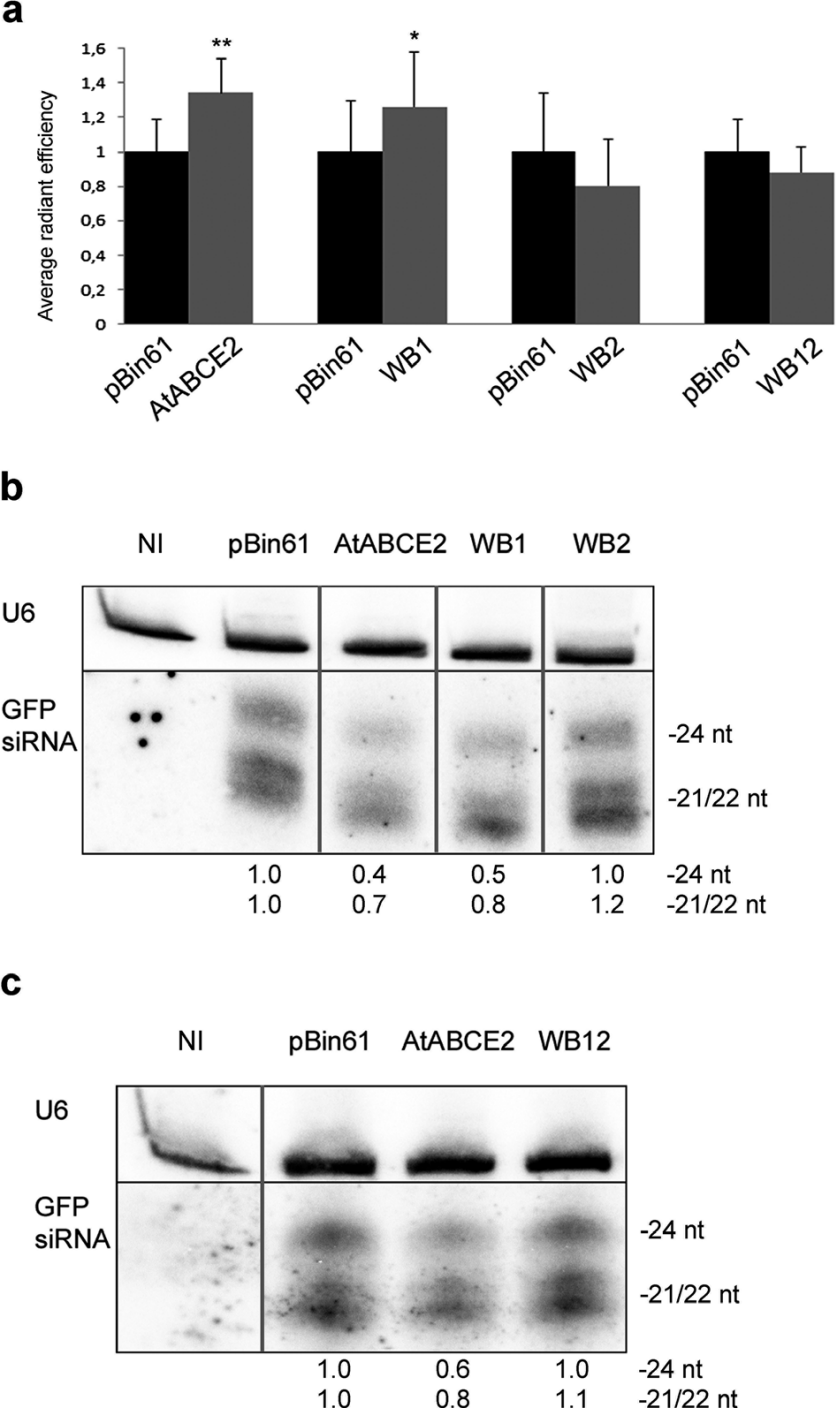
Suppression of local GFP RNA silencing by WB1, WB2 and WB12 mutants of AtABCE2. (a) *Agrobacterium tumefaciens* harbouring pBin61‐GFP was co‐infiltrated with *A. tumefaciens* carrying the pBin61‐AtABCE2 mutant or pBin61‐AtABCE2 wt into one half of 16c *Nicotiana benthamiana* leaf blade (indicated as WB1, WB2, WB12 and AtABCE2). The other half was infiltrated with GFP/pBin61 agrobacteria mixture (indicated as pBin61). At 5 dpi, the leaves were detached and subjected to *in vivo* imaging of GFP. Average of fluorescent radiant efficiency was measured in the infiltrated patches and compared in each leaf separately. The means of GFP expression were compared between pBin61 and AtABCE2 (n = 25), pBin61 and WB1 (n = 24), pBin61 and WB2 (n = 28), pBin61 and WB12 (n = 24). Obtained results were statistically analysed with a paired two‐tail *t‐*test (*P < 0.05, **P < 0.01). Standard errors are indicated as error bars. (b), (c) Total RNA was extracted from agroinfiltrated patches and analysed by Northern blot. GFP‐specific siRNAs were detected using [α‐^32^P] UTP‐labelled antisense GFP transcript. U6 snRNA is shown as a loading control (indicated as U6). The levels of GFP siRNAs (24 and 21/22 nt long), quantified with ImageQuant TL, were normalized to U6 and compared with pBin61. NI indicates non‐infiltrated leaf. The lanes were cropped from the same blots. Intact membranes are presented in Figure S2b,c.

Next, we explored how substitution of key residues in WB motifs (Fig. 1) shown to be involved in ATP hydrolysis in the case of aABCE1, affect the suppressor function of AtABCE2. The mutants WB1 (E242Q) and WB2 (E493Q), with putatively affected ATP hydrolysis in site I and site II, respectively, showed different suppression efficiency. GFP fluorescence intensity was significantly stronger in the leaf patches infiltrated with GFP/WB1 agrobacteria mixture compared to GFP/pBin61 (*P* = 0.0271) (Fig. 4a). This result was consistent with the detected reduced siRNA accumulation (Fig. 4b) and implies that WB1 suppresses local RNA silencing. Moreover, the WB1 mutant was able to interfere with the spread of systemic silencing, although less efficiently than AtABCE2 wt: 56% of plants infiltrated with this mutant were systemically silenced at 14 and 21 dpi, showing statistically significant difference from the empty vector (Table 2). In the leaf patches infiltrated with GFP/WB2 we detected low GFP fluorescence levels, consistent with abundant GFP siRNAs (Fig. 4a, b). In the case of the WB2 mutant, 71% and 79% of the infiltrated plants showed systemic silencing at 14 and 21 dpi, respectively. At 14 dpi, the difference between pBin61 and WB2 was significant (*P* = 0.04301) and the visible silencing (red tissue) was less extensive in the case of the mutant. However, at 21 dpi this statistical significance was lost (Table 2, Fig. 3). These results indicate that in contrast to WB1, the WB2 mutant failed to suppress GFP RNA silencing at both local and systemic levels. We also tested the double mutant, named WB12 (E242Q and E493Q), which possesses arrested closed conformation with both ATP molecules occluded in the interface between the NBDs. GFP expression was strongly reduced in the leaf patches infiltrated with WB12, and GFP siRNA levels were as high as in the case of pBin61 (Fig. 4a,c). Further, we observed the spread of systemic silencing in 79% of the plants infiltrated with GFP/WB12, showing that the simultaneous mutation of both WB motifs affects not only local but also systemic silencing suppression (Table 2).

### Suppression of GFP RNA silencing by AtABCE2∆FeS and AtABCE2∆HLH mutants

**Fig. 5:**
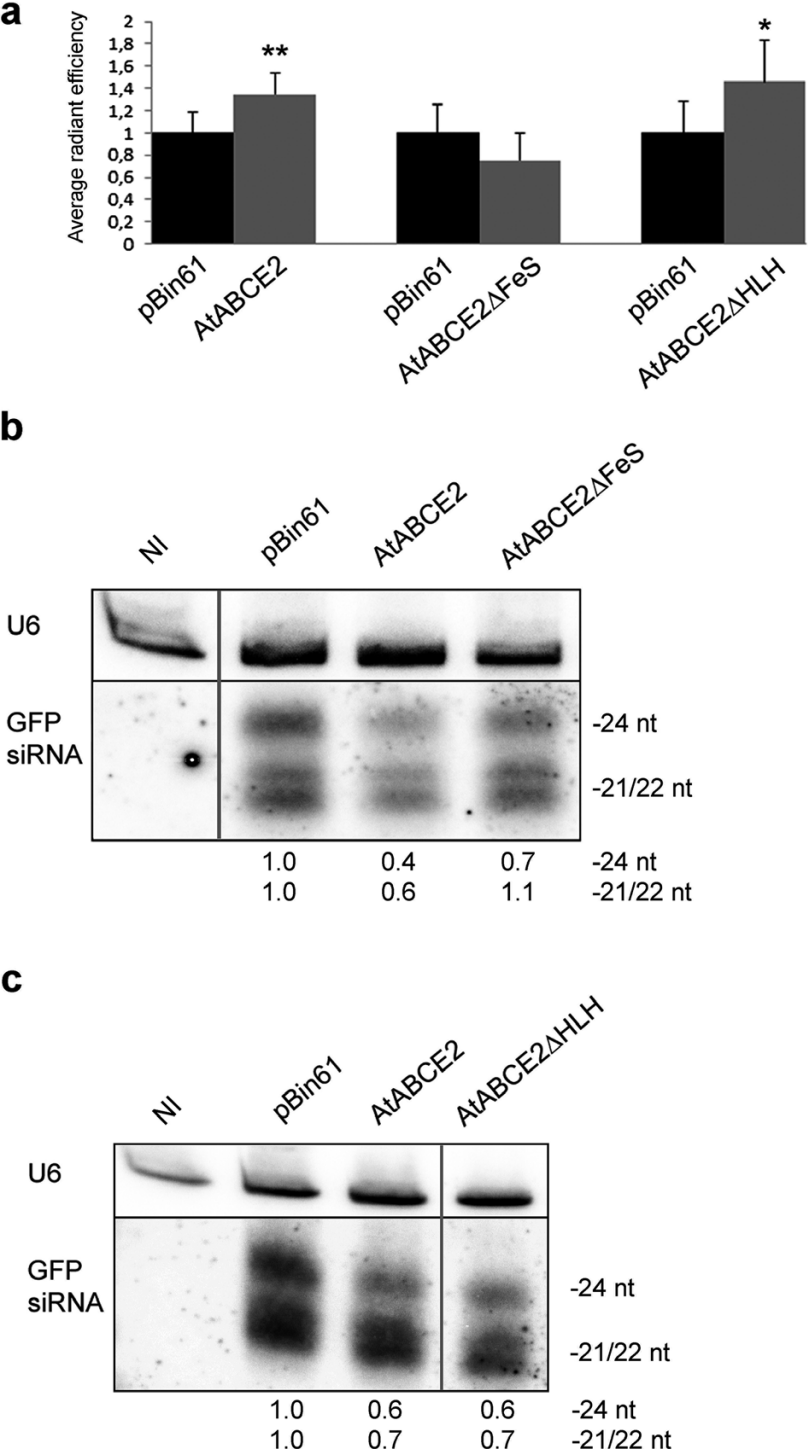
Suppression of local GFP RNA silencing by AtABCE2∆FeS and AtABCE2∆HLH mutants. (a) *Agrobacterium tumefaciens* harbouring pBin61‐GFP was co‐infiltrated with *A. tumefaciens* carrying the pBin61‐AtABCE2 mutant or pBin61‐AtABCE2 wt into one side of 16c *Nicotiana benthamiana* leaf blade (indicated as AtABCE2∆HLH, AtABCE2∆FeS and AtABCE2), and the other side was infiltrated with *A. tumefaciens* harbouring pBin61‐GFP together with *A. tumefaciens* carrying the empty vector (indicated as pBin61). At 5 dpi, the GFP fluorescence in the agroinfiltrated patches was measured with an *in vivo* imaging system. The values obtained from each leaf were compared separately. The data obtained from pBin61 and AtABCE2 (n = 25), pBin61 and AtABCE2∆FeS (n = 27), pBin61 and AtABCE2∆HLH (n = 24) pairs were statistically analysed with a paired two‐tail *t‐*test (*P < 0.05, **P < 0.01). Standard errors are indicated as error bars. (b), (c) Total RNA, extracted from agroinfiltrated patches was analysed by Northern blotting. The [α‐^32^P] UTP‐labelled antisense GFP transcript was used for hybridisation of GFP siRNAs. U6 snRNA was detected as equal RNA loading control (indicated as U6). The levels of GFP siRNAs (24 and 21/22 nt long), quantified with ImageQuant TL, were normalized to U6 and compared with pBin61. NI indicates non‐infiltrated leaf tissue. The lanes were cropped from the same blots. Intact membranes are presented in Figure S2d,e.

Finally, we analysed mutants comprising deletions of AtABCE2 potential interaction sites, namely the FeS cluster domain (AtABCE2∆FeS) and the HLH insertion in NBD1 (AtABCE2∆HLH). We found that in the presence of the AtABCE2∆FeS mutant, GFP expression levels in the infiltrated leaves were as low as in the case of pBin61, and this was in agreement with abundant levels of GFP siRNAs (Fig. 5a,b). In addition, AtABCE2∆FeS was not able to suppress systemic silencing (76% and 79% of the plants were silenced at 14 and 21 dpi, respectively) (Table 2). Exploring the importance of the HLH insertion for AtABCE2 RNA silencing suppression capacity, we found that at the local level it does not play a role: GFP expression levels were significantly higher in the leaf patches infiltrated with GFP/AtABCE2∆HLH compared to GFP/pBin61 (*P* = 0.0304), similar to AtABCE2 wt *versus* pBin61 (Fig. 5a). Consistently, GFP siRNA levels were strongly reduced in the presence of AtABCE2∆HLH, supporting the suppression of local silencing (Fig. 5c). Although AtABCE2∆HLH suppressed local GFP silencing, it was unable to do this at the systemic level: 79% and 83% of the plants were systemically silenced at 14 and 21 dpi, respectively (Table 2).

## DISCUSSION

There have been many studies of ABCE1 as a translational and ribosome recycling factor in archaea, yeast and animals (Dong *et al.,* 2004; Kispal *et al.,* 2005; Yarunin *et al.,* 2005; Chen *et al.,* 2006; Pisarev *et al.,* 2010; Nürenberg & Tampé, 2013; Nürenberg‐Goloub *et al.,* 2018; Gerovac & Tampé, 2019). However, little is known about ABCE proteins in plants. We have previously demonstrated that AtABCE2 and human ABCE1 act as RNA silencing suppressors, although the structural basis as well as the mechanism of suppression have remained elusive so far (Sarmiento *et al.,* 2006; Kärblane *et al.,* 2015). In this research, we report the structural requirements of AtABCE2 for the suppression of transgene RNA silencing in GFP‐*N. benthamiana* plants. Within the framework of mutational analysis, we examined the importance of ATP binding and hydrolysis for the suppressor function of AtABCE2. Mutants lacking the potential interaction sites, the FeS cluster domain and the HLH motif, were also explored.

We found that substitution of key residues involved in ATP binding at either the first (WA1C2 mutant) or the second (WA2C1) active centre strongly affects AtABCE2 suppression ability. At the local level, a higher accumulation of GFP siRNAs was observed in tissues expressing WA1C2 and WA2C1 compared to the AtABCE2 wt. These results are consistent with low GFP fluorescence levels, comparable with those observed in tissues transformed with the empty plasmid. Furthermore, the mutants could not suppress the spread of GFP silencing into the upper leaves. Based on these findings, we report that ATP binding at the first and the second active centre is necessary for the suppression activity of AtABCE2 at local as well as at systemic levels. Interestingly, WA12C12, harbouring simultaneously mutated ATP binding site I and site II, suppressed systemic GFP silencing but had reduced suppression activity at the local level. We cannot rule out the possibility that a disruption of key residues in both active sites leads to drastic conformational changes in the ATPase core that provides an alternative mode of ATP binding. This issue needs further investigation.

Next, we questioned whether ATP hydrolysis is necessary for AtABCE2 suppressor function. We found that an AtABCE2 mutant with affected ATP hydrolysis in site I (WB1) suppresses RNA silencing at the local level, similar to the AtABCE2 wt. WB1 is also able to suppress systemic silencing, however less efficiently than the AtABCE2 wt. In contrast, the WB2 mutant, defective in hydrolysis in site II, could not suppress both local and systemic GFP silencing. The loss of function was also observed in the case of the double mutant (WB12). These results indicate that ATP hydrolysis in site II is necessary and sufficient for AtABCE2 suppression activity, while ATP hydrolysis in site I does not seem to be critical for this function. The fact that the two ATP binding sites play distinct roles and have different ATPase activities was previously established for archaeal ABCE1 in the context of ribosome recycling (Barthelme *et al.,* 2011; Nürenberg‐Goloub *et al.,* 2018), and therefore it was not entirely surprising to observe a similar effect for another function of ABCE1. A recent article on the role of aABCE1 in ribosome recycling during canonical termination of translation provided data on timing and conformational changes in aABCE1 during each step (Nürenberg‐Goloub *et al.,* 2018). There is an obvious parallel to our observations in the case of RNA silencing suppression by AtABCE2. To bind the post‐termination complex, site II in aABCE1 must occlude ATP, thereby strongly stimulating ATP binding and hydrolysis in site I. ATP binding at site I is crucial for splitting the ribosomal subunits. After final and stable ATP binding in both sites, aABCE1 remains bound to the 40S subunit. For further dissociation from the ribosomal subunit, aABCE1 must hydrolyse ATP. At this step, ATP hydrolysis in site II is critical, but not in site I, according to the results published by Nürenberg‐Goloub *et al.* (2018). In our research, we observed that only WB1, the mutant with affected ATP hydrolysis in site I, is able to suppress GFP RNA silencing at the local and systemic levels. The mutants lacking ATP binding activity at either site I or site II, as well as the mutants with impaired ATP hydrolysis at both sites or only at site II, have no suppression capacity.

Beside the ATPase core, ABCE1 possesses a unique (among ABC ATPases) FeS cluster domain and a no less intriguing HLH motif in NBD1. According to multiple research on ABCE1 in archaea, yeast and mammalian cells, the FeS cluster domain was found to be indispensable for ABCE1 function in translation and ribosome recycling. It was reported that the FeS cluster domain directly interacts with the ribosomal subunits and recycling factors eRF1 and Pelota, which are involved in canonical and mRNA surveillance‐triggered termination of translation, respectively. During ribosome recycling, the FeS cluster domain undergoes extreme relocation upon ATP binding and thereby drives ribosome splitting (Pisareva *et al.,* 2011; Becker *et al.,* 2012; Kiosze‐Becker *et al.,* 2016). In addition, through chemical cross‐linking and mass spectrometry, multiple contacts were detected between the HLH motif and S24e ribosomal protein, as well as between the FeS cluster domain and S12 in the post‐recycling complex (Kiosze‐Becker *et al.,* 2016; Heuer *et al.,* 2017).

Our interest was to find out whether the FeS cluster domain or the HLH motif also contribute to the AtABCE2 suppressor function. We found that the AtABCE2∆FeS mutant failed to suppress local as well as systemic silencing, indicating the indispensable role of this domain for AtABCE2 suppressor activity. Despite the fact that the FeS cluster domain forms an autonomous rigid entity, the complex of two NBDs directly regulates its relocation through an ATP binding/hydrolysis cycle (Kiosze‐Becker *et al.,* 2016; Heuer *et al.,* 2017). According to our results, the FeS cluster domain, and likely its translocation, are necessary for the suppression activity of AtABCE2. The effect of HLH full deletion in other systems has not previously been studied. Here we demonstrate that deleting the entire HLH motif does not inhibit AtABCE2 suppressor function, since the AtABCE2∆HLH mutant was able to suppress local GFP silencing. However, the deletion seems to affect the suppression of systemic silencing, indicating the somewhat supportive role of the HLH motif. One possible explanation might be that the HLH motif allows stronger interaction with factors affecting RNA silencing. Another possible interpretation would be to consider it as an important factor for ATPase core stability during the ATP binding/hydrolysis cycle.

Importantly, other RNA degradation mechanisms in eukaryotes, apart from RNA silencing, are required to provide RNA homeostasis in the cells. These are referred to as mRNA surveillance pathways: nonstop decay, no‐go decay and nonsense‐mediated decay. These processes are coupled with translation and strongly depend on the recycling of ribosomes (Graille & Séraphin, 2012). In *Drosophila*, mammalian cells and in yeast, ABCE1 mediates, in a concerted way with Pelota/Dom34 and Hbs1, the dissociation of ribosomes, which are stalled because of aberrant mRNAs. As a result, defective mRNAs can be eliminated by the Ski/exosome complex and Xrn1 exonuclease through either NSD, in the case of stop codon‐less mRNA, or NGD, if mRNA contains elongation inhibiting features (Pisareva *et al.,* 2011; Kashima *et al.,* 2014; Hashimoto *et al.,* 2017; Navarro‐Quiles *et al.,* 2018). In plants, the data concerning NSD and NGD are still largely missing. However, it was recently shown that *A. thaliana* encodes two Pelota paralogues: Pelota1 is a positive regulator of NSD and NGD, while Pelota2 suppresses both RNA decay pathways (Szádeczky‐Kardoss *et al.,* 2018a; Szádeczky‐Kardoss *et al.,* 2018b). It is not yet certain whether plant ABCE proteins interact with Pelota and function in ribosome recycling, although the high conservation of this process makes this very likely. Moreover, experiments involving *Cardamine hirsuta* ABCE2 (also named ChRLI2) revealed it as an important regulator of leaf growth and proposed, for the first time, a link between ABCE2 and ribosomes in plants (Kougioumoutzi *et al.,* 2013). Notably, mRNA surveillance pathways were found to compete with RNA silencing machinery for the substrate (Christie *et al.,* 2011; Liu & Chen, 2016; Szádeczky‐Kardoss *et al.,* 2018a; Kim *et al.,* 2019). Because of the above results, we suggest that AtABCE2 might suppress RNA silencing *via* positive regulation of mRNA surveillance mechanisms. However, we cannot rule out another scenario, by which AtABCE2 directly interacts with components of RNA silencing pathways. We have previously identified a number of potential interactors for human ABCE1, which might contribute to its suppressor function and have putative homologues in *A. thaliana* (Kärblane *et al.,* 2015).

Taken together, mutational analysis results show that ATP binding and hydrolysis are important for AtABCE2 suppressor function. The FeS cluster domain likely mediates interactions with partners and is critical for the suppression of RNA silencing. High similarity between the structural requirements for the AtABCE2 suppressor function and those for aABCE1 role in ribosome recycling, crucial for mRNA surveillance, leads us to hypothesize that AtABCE2 could support RNA degradation pathways that compete with RNA silencing and therefore act as a negative regulator of silencing. The interactions of the AtABCE2 protein with the translational machinery, as well as with RNA silencing factors in plants, remain to be investigated.

## AUTHOR CONTRIBUTIONS

Conceptualization, J.M., E.T. and C.S.; methodology, J.M., P.E. and C.S.; formal analysis, J.M. and C.S.; investigation, J.M., S.M. and C.S.; data curation, J.M.; writing of original draft preparation, J.M.; writing for review and editing, S.M., P.E., E.T. and C.S.; visualization, J.M.; supervision, C.S.; project administration, C.S.; funding acquisition, E.T and CS. All authors have read and agree to the published version of the manuscript.

## FUNDING

This research was funded by the Estonian Ministry of Education and Research (institutional research funding IUT193). This work was partially supported by the ‘TUT Institutional Development Program for 2016–2022’, the Graduate School in Biomedicine and Biotechnology, which received funding from the European Regional Development Fund under programme ASTRA 2014‐2020.4.01.16‐0032 in Estonia.

## CONFLICTS OF INTEREST

The authors declare that they have no conflict of interest.

## Supporting information


**Figure S1**. Representation of *in vivo* imaging of GFP.
**Figure S2**. The full‐length Northern blots corresponding to the figures in the main text.
**Table S1**. Results of systemic silencing assay for nine independent experiments.
**Table S2**. Statistical analysis for the systemic silencing assay.
