# Supplementary material for: Mutational analysis of Arabidopsis thaliana ABCE2 identifies important motifs for its RNA silencing suppressor function
Source: Plant Biol (Stuttg). 2020 Nov 29;23(1):21–31. doi: 10.1111/plb.13193 (PMC7839781; doi:10.1111/plb.13193)
Supplement: Supplementary file 1 — Figure S1. Representation of in vivo imaging of GFP. Figure S2. The full‐length Northern blots corresponding to the figures in the main text. Table S1. Results of systemic silencing assay for nine independent experiments. Table S2. Statistical analysis for the systemic silencing assay. [file PLB-23-21-s001.docx]

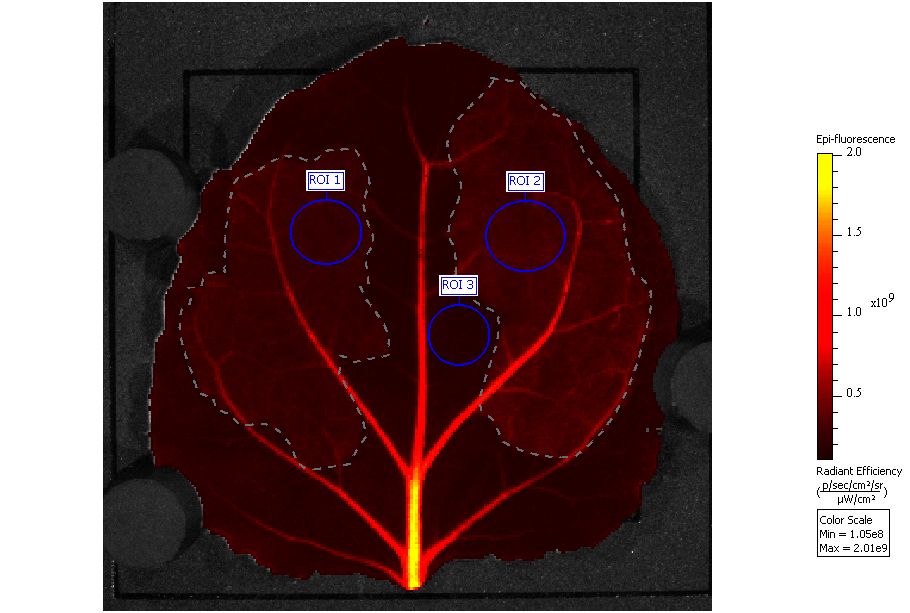


**Figure S1. Representation of *in vivo* imaging of GFP for quantifying the suppression of local GFP RNA silencing.**

The images of infiltrated leaves were acquired using *in vivo* imaging system (IVIS Lumina II (Caliper Life Sciences) equipped with GFP-specific filter set (465 nm excitation filter and GFP emission filter). The obtained pseudocolour images contain numerical data on the amount of light emission in each pixel. The colour bar on the right shows radiant efficiency of epi-fluorescence and is related to the data from the image. The average radiant efficiency of GFP, which has no units and indicates the ratio of emitted to incident light in the representative area of each patch (indicated as ROI1 and ROI2), was quantified and normalized to the background (ROI3). The infiltrated patches are circled with dashed lines.

**

**

**Figure S2.** The full length Northern blots corresponding to the following figures from the main text: **(a)** Figure 2b, **(b)** Figure 4b, **(c)** Figure 4c, **(d)** Figure 5b, **(e)** Figure 5c. The unnamed lanes were not included in this analysis. [γ-^32^P] ATP-labelled 30 nt DNA oligo was used as a size marker.

**Table S1.** Percentages of systemically silenced plants at 14 and 21 dpi. Results obtained from 9 independent experiments. Four to six experiments were carried out for each AtABCE2 mutant**.**

| **experiment** | **Exp 1** | | **Exp 2** | | **Exp 3** | | **Exp 4** | | **Exp 5** | | **Exp 6** | | **Exp 7** | | **Exp 8** | | **Exp 9** | |
| --- | --- | --- | --- | --- | --- | --- | --- | --- | --- | --- | --- | --- | --- | --- | --- | --- | --- | --- |
| **(number of plants)** | **(7)** | | **(7)** | | **(7)** | | **(5)** | | **(5)** | | **(7)** | | **(5)** | | **(5)** | | **(7)** | |
|  | **14 dpi** | **21 dpi** | **14 dpi** | **21 dpi** | **14 dpi** | **21 dpi** | **14 dpi** | **21 dpi** | **14 dpi** | **21 dpi** | **14 dpi** | **21 dpi** | **14 dpi** | **21 dpi** | **14 dpi** | **21 dpi** | **14 dpi** | **21 dpi** |
| pBin61 | 86% | 100% | 86% | 100% | 71% | 71% | 100% | 100% | 100% | 100% | 100% | 100% | 100% | 100% | 100% | 100% | 86% | 86% |
| AtABCE2 | 14% | 14% | 43% | 43% | 29% | 29% | 40% | 40% | 80% | 80% | 43% | 43% | 80% | 80% | 40% | 40% | 43% | 71% |
| WA1C2 | 57% | 71% |  |  |  |  |  |  | 100% | 100% |  |  |  |  | 80% | 80% | 57% | 71% |
| WA2C1 | 43% | 57% |  |  |  |  |  |  | 100% | 100% |  |  |  |  | 80% | 80% | 86% | 100% |
| WA12C12 | 57% | 57% | 57% | 71% | 29% | 29% |  |  | 100% | 100% |  |  |  |  | 60% | 60% | 29% | 29% |
| WB1 |  |  | 43% | 43% |  |  | 60% | 60% | 100% | 100% |  |  | 60% | 60% | 40% | 40% | 43% | 43% |
| WB2 |  |  | 57% | 71% |  |  | 40% | 40% | 100% | 100% |  |  | 80% | 100% | 80% | 80% | 71% | 86% |
| WB12 | 71% | 71% |  |  |  |  |  |  | 100% | 100% |  |  |  |  | 80% | 80% | 71% | 71% |
| AtABCE2∆FeS |  |  |  |  |  |  | 60% | 80% | 100% | 100% | 71% | 71% | 100% | 100% | 60% | 60% | 71% | 71% |
| AtABCE2∆HLH | 86% | 86% |  |  |  |  | 80% | 80% | 80% | 100% |  |  |  |  | 80% | 80% | 71% | 71% |

**Table S2.** Statistical analysis performed by JMP software for systemic silencing assay

| **14 dpi** |  |  |
| --- | --- | --- |
| Test | ChiSquare | Prob>ChiSq |
| Likelihood Ratio | 41.556 | <0.0001 |
| Pearson | 39.85 | <0.0001 |
|  |  |  |
| **Fisher's Exact Test** |  |  |
|  | Pvalue (2-tail) | **FDR Adj PValue** |
| pBin61-AtABCE2 | 1.45E-07 | 1.30E-06 |
| pBin61-WA12C12 | 4.85E-05 | 0.00022 |
| pBin61-WB1 | 0.0002 | 0.00059 |
| pBin61-WB2 | 0.01912 | 0.04301 |
| pBin61-WA1C2 | 0.0377 | 0.06786 |
| pBin61-AtABCE2∆FeS | 0.07206 | 0.10347 |
| pBin61-WA2C1 | 0.08048 | 0.10347 |
| pBin61-WB12 | 0.16191 | 0.17666 |
| pBin61-AtABCE2∆HLH | 0.17666 | 0.17666 |
|  |  |  |
| **21 dpi** |  |  |
| Test | ChiSquare | Prob>ChiSq |
| Likelihood Ratio | 48.959 | <0.0001 |
| Pearson | 46.832 | <0.0001 |
|  |  |  |
| **Fisher's Exact Test** |  |  |
|  | Pvalue (2-tail) | **FDR Adj PValue** |
| pBin61-AtABCE2 | 3.68E-08 | 3.31E-07 |
| pBin61-WA12C12 | 1.23E-05 | 5.15E-05 |
| pBin61-WB1 | 1.72E-05 | 5.15E-05 |
| pBin61-WB2 | 0.0394 | 0.06578 |
| pBin61-AtABCE2∆FeS | 0.0394 | 0.06578 |
| pBin61-WA1C2 | 0.05116 | 0.06578 |
| pBin61-WB12 | 0.05116 | 0.06578 |
| pBin61-AtABCE2∆HLH | 0.11764 | 0.13235 |
| pBin61-WA2C1 | 0.19062 | 0.19062 |
